# The Role of Microbiota in Ovarian Cancer: Implications for Treatment Response and Therapeutic Strategies

**DOI:** 10.3390/cells14221813

**Published:** 2025-11-19

**Authors:** Jose-Ramon Blanco, Rosa del Campo, José Avendaño-Ortiz, Mariano Laguna-Olmos, Amancio Carnero

**Affiliations:** 1 Hospital Universitario San Pedro, 26006 Logroño, Spain; mlaguna@riojasalud.es; 2 Centro de Investigación Biomédica de La Rioja (CIBIR), 26006 Logroño, Spain; 3 Grupo de Investigación en Cuidados y Salud (GRUPAC), de la Universidad de La Rioja, 26006 Logroño, Spain; 4 Centro de Investigación Biomédica en Red Enfermedades Infecciosas (CIBERINFEC), 28029 Madrid, Spain; joseavenort@gmail.com; 5 Facultad Ciencias de la Salud, Universidad de La Rioja, La Rioja, 26006 Logroño, Spain; 6 Servicio de Microbiología, Hospital Universitario Ramón y Cajal and Instituto Ramón y Cajal de Investigación Sanitaria (IRYCIS), 28034 Madrid, Spain; 7 Instituto de Biomedicina de Sevilla (IBIS)/HUVR/CSIC, Universidad de Sevilla, 41013 Sevilla, Spain; acarnero-ibis@us.es; 8 CIBER de Cancer (CIBERONC), Instituto de Salud Carlos III, 28029 Madrid, Spain

**Keywords:** gut microbiota, ovarian cancer, biomarker, prognosis

## Abstract

Cancer remains a global health challenge (18.1 million new cases in 2020), with incidence projected to reach 28 million within two decades. Ovarian cancer (OC) is the deadliest gynecologic malignancy, usually diagnosed at advanced stages and with poorly understood etiology. Emerging evidence implicates reproductive tract and gut microbiota in OC biology. Microbiota shape carcinogenesis via turnover, immunity, and metabolism; dysbiosis promotes DNA damage, inflammation, and carcinogenic metabolites, engaging multiple hallmarks of cancer. In OC, microbes may reach tumors by local ascent, translocation, or hematogenous spread, originating from vagina, upper reproductive tract, peritoneal fluid, or gut. *Lactobacillus*-dominant vaginal communities support mucosal integrity, whereas anaerobes disrupt barriers, increase inflammation, and correlate with OC risk; mouse models show vaginal dysbiosis accelerates tumor progression. Distinct microbial profiles in upper reproductive sites and peritoneal fluid associated with immune remodeling. Gut dysbiosis drives barrier loss, immune imbalance, and estrogen reactivation. Microbial metabolites (lipopolysaccharides, short-chain fatty acids) modulate oncogenic pathways, altering epithelial–mesenchymal transition, immune evasion, and drug resistance. Across cohorts, OC tissues and fluids show Pseudomonadota/Bacteroidota enrichment and *Akkermansia* depletion; fecal microbiota from OC patients accelerates tumor growth in mice, whereas *Akkermansia* supplementation restores antitumor immunity. Antibiotic exposure and platinum resistance associate with reduced diversity and expansion of lactate-producing taxa. Microbiome-informed interventions–diet, probiotics/postbiotics, fecal microbiota transfer, and selective antibiotics–may augment chemotherapy and immunotherapy. Overall, the microbiome is a modifiable determinant of OC risk, progression, and treatment response, warranting rigorous, standardized, multi-omics studies.

## 1. Introduction

In 2020, approximately 18.1 million new cancer cases were reported worldwide, with projections approaching 28.0 million within the next two decades [1]. Ovarian cancer (OC) is the eighth most common malignancy in women and the deadliest gynecologic cancer; nearly 80% of OC cases present at advanced stages, and outcomes remain suboptimal despite therapeutic advances [2,3,4]. OC is heterogeneous, about 90% are epithelial, of which 70–80% are high-grade serous; less common histotypes (endometrioid, clear cell, low-grade serous, mucinous, carcinosarcoma) differ in genetics and therapeutic sensitivity [2,3]. Although its etiopathogenesis remains incompletely defined, accumulating evidence implicates the gastrointestinal and female reproductive-tract microbiota in gynecological tumor initiation, progression, and therapeutic response [5].

Here, we synthesize current evidence on microbiota-OC interactions and propose a unified mechanistic framework integrating immune, metabolic, and endocrine axes. Distinct from prior reviews, we deliver a mechanism-anchored, cross-compartment synthesis with explicit contamination control and actionable trial-design guidance. We then appraise clinical and translational implications for treatment response and resistance and evaluate microbiota-directed interventions—including probiotics and fecal microbiota transplantation (FMT).

This narrative, mechanism-focused review surveyed PubMed, Scopus, and Web of Science through September 2025 using combinations of “ovarian cancer”, “microbiome/microbiota”, “dysbiosis”, “chemotherapy”, “immunotherapy”, “estrobolome”, “probiotics”, and “FMT”. We included peer-reviewed, English-language original studies (preclinical and clinical) employing 16S rDNA gene sequencing or shotgun metagenomics, with complementary metabolomic/transcriptomic analyses, across tumor, peritoneal, fecal, and vaginal specimens; reviews/editorials informed background only. Evidence was synthesized by mechanistic plausibility, reproducibility across cohorts, and translational relevance, with attention to contamination control and low-biomass constraints. We followed PRISMA principles for transparency; no quantitative meta-analysis was performed.

## 2. Mechanistic Framework: Microbiota–Host Crosstalk in Ovarian Carcinogenesis

The human microbiota modulates host physiology by shaping immune responses, epithelial integrity, metabolism, and endocrine signaling [6,7,8,9,10,11]. Garret [12] outlined three principal mechanisms by which microbes influence carcinogenesis: (1) regulation of host cell proliferation and death, (2) modulation of immunity, and (3) alteration of the metabolism of dietary constituents and host-derived factors.

Microbial dysbiosis can promote tumorigenesis through genotoxicity and chronic inflammation. Mechanistically, bacteria and their products induce DNA damage, impair DNA repair, and generate carcinogenic metabolites; in parallel, barrier disruption sustains mucosal and systemic inflammation, amplifying oncogenic pathways [13,14,15,16,17]. These processes intersect with multiple “hallmarks of cancer”, including sustained proliferative signaling, resistance to cell death, replicative immortality, angiogenesis, and immune evasion [18].

Innate immune pattern-recognition provides a direct link between microbial signals and tumor behavior. Engagement of toll-like receptors (TLR) by microbe-associated molecular patterns, such as lipopolysaccharide (LPS), activates NF-κB, MAPK/ERK, JAK/STAT, and PI3K-Akt signaling cascades that elicit pro-tumorigenic cytokine networks (e.g., IL-6, IL-8, TNF-α, VEGF) and epithelial–mesenchymal transition (EMT), thereby fostering survival, invasion, and chemoresistance [19,20,21,22,23,24,25]. In OC, TLR4 activation by LPS or paclitaxel increases inflammatory mediators and resistance to cell death, while TLR4 silencing restores chemosensitivity [23,24,25].

Converging evidence implicates a gut-vaginal microbiome axis in ovarian carcinogenesis–via estrogen re-metabolism, pro-inflammatory NF-κB/STAT3 signaling, and DNMT (DNA Methyltransferases)-driven epigenetic repression–supporting a multi-omics, machine-learning diagnostic strategy to enhance early detection [26].

An endocrine–microbiome axis further integrates microbial activity with hormone-dependent tumor biology. The estrobolome, a set of β-glucuronidase genes that deconjugate estrogens and enable reabsorption, regulates enterohepatic recycling of estrogens, influencing systemic exposure [27,28,29]. Dysbiosis-associated estrobolome overactivity, often linked to obesity and metabolic dysfunction, has been implicated in estrogen-driven neoplasia and may contribute to OC risk phenotypes, particularly non-serous histology that correlates with elevated levels of unconjugated estradiol [30,31,32,33].

Using an in vitro colonic fermentation model simulating female and male gut environments, hormonal fluctuations across the menstrual cycle were shown to modulate microbial diversity and the abundance of key taxa. In the female model, elevated progesterone and estrogen levels were associated with shifts in Bacteroidota, Bacillota, and Pseudomonadota, as well as shifts in microbial metabolite profiles [34]. These findings highlight the capacity of sex hormones to dynamically shape gut microbiota composition and function, reinforcing endocrine-microbial crosstalk as a mechanistic link in hormone-dependent tumorigenesis.

**Figure 1:**
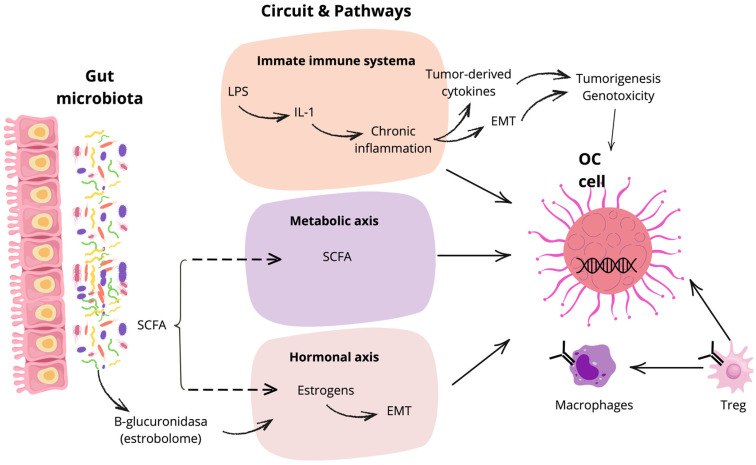
Immune, metabolic, and hormonal axes linking the gut microbiome to ovarian tumorigenesis. Microbial LPS promotes IL-1-driven chronic inflammation, tumor-derived cytokines foster EMT and genotoxicity; while SCFAs rewire immunometabolism, and estrobolome-derived estrogens cooperate to promote EMT programs in ovarian cancer cells, macrophage polarization, and Treg accrual. EMT: epithelial–mesenchymal transition; IL: interleukin; LPS: lipopolysaccharide; OC: ovarian cancer; SCFA, short-chain fatty acid.

Microbial metabolites act as bioactive mediators with epigenetic and immunometabolic effects. Short-chain fatty acids (SCFAs)–acetate, propionate, and butyrate–are produced by fermentation of dietary fiber and regulate histone acetylation, T cell differentiation, and epithelial renewal [35,36,37,38,39,40,41,42]. In OC models, butyrate functions as a histone deacetylase inhibitor, inducing apoptosis, suppressing proliferation, and reprogramming epigenetic states [39,43,44,45,46,47]. Beyond SCFAs, secondary bile acids, cadaverine, and enterolignans modulate signaling pathways relevant to tumor progression [35,36,37,38,39,40]. Notably, taxon-specific effects can diverge from expected metabolic profiles; for example, members of the family *Lachnospiraceae*, a butyrate-producing family implicated in estrogen metabolism, have paradoxically been associated with increased OC risk in some cohorts [48] (Figure 1).

## 3. Microbial Niches and Routes of Dissemination in OC

**Figure 2:**
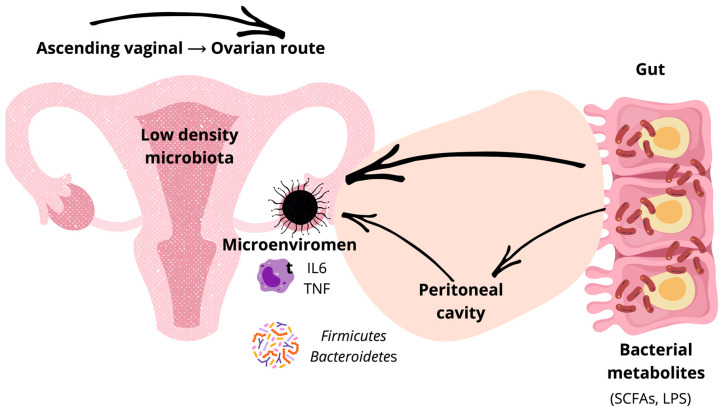
Bidirectional gut–peritoneal–ovarian communication and microbial metabolite flux in ovarian cancer. Low-density upper-female-tract microbiota, peritoneal cavity exposure, and gut-derived SCFAs/LPS converge to shape inflammatory mediators (IL-6, TNF) and the ovarian tumor microenvironment, consistent with ascending routes and metabolite crosstalk. IL: interleukin; LPS: lipopolysaccharide; SCFA, short-chain fatty acid; TNF: tumor necrosis factor.

Microbes associated with tumors can arise from local overgrowth, translocation across disrupted mucosal barriers, contiguous spread from adjacent tissues, or hematogenous dissemination [49]. In OC, plausible sources include the lower reproductive tract (vagina and cervix), the upper reproductive tract (uterus, fallopian tubes, ovaries, and peritoneal fluid), the gut, and the bloodstream [49]. However, whether—and to what extent—the ovarian-tumor microbiota varies across histopathological subtypes remains unclear. Defining these routes is essential to understanding how microbial communities shape tumor microenvironments and treatment responses (Figure 2).

### 3.1. Lower Reproductive Tract

Vaginal and cervical communities are structured into community state types that are frequently *Lactobacillus*-dominant in reproductive-age women; lactic acid-mediated acidification maintains a low vaginal pH that restricts pathogen colonization [50,51,52]. Dysbiosis, characterized by *Lactobacillus* spp. depletion with anaerobic overgrowth, disrupts epithelial barriers, enhances mucosal permeability, and increases susceptibility to ascending infections and pelvic inflammatory disease (PID), and has been linked to persistent HPV infection and gynecological malignancies [52,53,54,55,56,57,58,59].

Mutations in the breast cancer gene (BRCA)1/2 substantially increase the risk of OC [60,61] and may influence host–microbiota–hormone cross-regulation that modulates susceptibility. Women carrying BRCA mutations exhibit depletion of *Lactobacillus* spp., associated with elevated luteal-phase progesterone levels that reduce vaginal glycogen availability [55]. As *Lactobacilli* rely on glycogen metabolism, this hormonal environment may contribute to their decline, and reduced *Lactobacillus* abundance could represent a mechanistic link between endocrine dysregulation and epithelial transformation [55].

Epidemiologically, severe PID associates with increased OC risk, plausibly through chronic inflammation and tissue remodeling [53,54,55]. However, current links between the vaginal microbiota and OC are largely correlational: the ascent hypothesis remains unproven, with substantial confounding (e.g., age, hormones, BRCA genotype). Longitudinal and mechanistic studies are therefore required to establish causality. Clinically, managing vaginal dysbiosis is a pragmatic target for primary prevention [62].

Progesterone’s role in OC is multifaceted and context-dependent. Epidemiologically and experimentally, it is generally regarded as protective, as positive nuclear progesterone receptor (nPR) expression correlates with improved clinical outcomes [63]. Transcriptomic analyses further show that *BRCA*-mutant fallopian tube epithelia most closely resemble high-grade serous ovarian carcinoma when sampled during the luteal phase, coinciding with peak progesterone concentrations [64]. In vitro, short-term exposure of OC cell lines to progestins reduces viability, suppresses proliferation, and induces cell death, consistent with progesterone’s tumor-suppressive actions [65].

### 3.2. Upper Reproductive Tract

Microbial DNA and viable bacteria have been detected in the uterus, fallopian tubes, ovaries, and peritoneal fluid, each harboring compositionally distinct communities in OC [66,67,68]. In the fallopian tubes, OC has been associated with reduced alpha-diversity, a higher Pseudomonadota-to-Bacillota ratio, and enrichment of *Acinetobacter*, *Sphingomonas*, and *Methylobacterium*, accompanied by immune transcriptional remodeling [67,69].

However, beyond microbial alterations, mechanistic studies indicate that nPR signaling itself can adopt pro-tumorigenic functions. In vitro and transgenic mouse models demonstrate that an imbalance of nPR isoforms can drive ovarian tumorigenesis [70]. Transcriptomic profiling of these models revealed proliferative gene signatures enriched in PI3K–AKT signaling, cell-cycle control, and DNA recombination pathways, mirroring molecular features of human endometrial and OC [71]. Notably, these tumors arose without engineered genetic mutations, implying that sustained nPR activation alone can initiate oncogenic transcriptional states.

Peritoneal fluid profiles also differ between OC and benign conditions and include gut-derived taxa that may reflect microbial translocation and systematic crosstalk [68,72].

These seemingly paradoxical findings—protective epidemiological associations versus oncogenic mechanistic data—underscore the context dependence of progesterone signaling. In *BRCA*-mutant carriers, elevated progesterone may contribute to *Lactobacillus* depletion, yet its causal role in OC risk remains uncertain [73]. Additional factors beyond progesterone likely influence *Lactobacillus* dynamics and epithelial susceptibility, reflecting a multifactorial interplay among hormonal signaling, microbial ecology, and tissue remodeling within the reproductive tract.

To disentangle progesterone’s dualistic actions, particularly in high-grade serous OC, future studies must account for nPR isoform complexity and its crosstalk with other steroid receptors, including the estrogen receptor and glucocorticoid receptor [74]. Understanding these multilayered interactions is essential to determine whether progesterone acts predominantly as an anti- or pro-tumorigenic factor across OC subtypes.

### 3.3. Gut and Systemic Sources

The colon harbors the most abundant and functionally diverse human microbiome [75,76], which regulates barrier integrity, immune crosstalk, and tumor-permissive environments [77]. Gut community structure differs between early and late OC stages and correlates with metabolic remodeling [78,79].

Murine xenograft studies show that antibiotic-induced dysbiosis can accelerate OC growth through macrophage activation and inflammatory cytokine release (IL-6, TNF-α) that promotes epithelial–mesenchymal transition, although findings are not universally replicated [80,81].

## 4. Stage- and Site-Specific Microbiota Alterations in OC: Mechanisms, Biomarkers, and Therapeutic Implications

Distinct microbial niches contribute to OC pathogenesis and progression. In murine OC models, overlapping vaginal and ovarian communities support lower-tract translocation; intravaginal antibiotics reduced tumor burden by around 40% and decreased aggressiveness without overt toxicity [82]. Separately, gut dysbiosis has been shown to accelerate OC via EMT and pro-inflammatory cytokines [80]. Together, these studies suggest site-specific microbial remodeling of the tumor microenvironment through convergent yet distinct mechanisms, revealing multiple therapeutic entry points.

Across cohorts, late-stage epithelial OC exhibits marked differences in gut community structure compared with early-stage disease, including dramatic fold-changes in specific taxa [78,83]. Reductions in *Akkermansia* are reported consistently; FMT from OC patients into tumor-bearing mice accelerates tumor growth, whereas *A. muciniphila* supplementation reverses this effect and enhances CD8^+^ T cell function via IFN-γ production [84,85].

Within Bacteroidota, alterations localize to Bacteroidales, with depletion of *Porphyromonadaceae* and *Rikenellaceae*, families associated with SCFA production and metabolic health, and enrichment of *Prevotella* spp. linked to gynecologic malignancies and coagulopathies [86,87,88,89,90]. Decreases in *Bifidobacterium* and *Bifidobacteriaceae* recur across studies [78,83,84].

Host–microbe crosstalk is reflected in tumor transcriptomes. OC tissues harbor bacterial phyla, often dominated by Pseudomonadota, and show enrichment of immune and cancer-related pathways, including JAK–STAT signaling and Th1/Th2 differentiation [91].

Mendelian randomization analysis provides orthogonal support for causal or bidirectional associations between gut taxa and OC risk, implicating genera such as *Dialister*, *Phascolarctobacterium*, *Bifidobacterium*, *Oscillibacter*, and others; however, many signals do not survive multiple-testing correction and should be interpreted cautiously [92,93,94,95].

## 5. Intratumoral Microbiota in OC: Distinct Signatures and Oncogenic Pathways

Multiple studies report altered diversity and taxonomic composition within OC tissue compared with adjacent normal or benign tissue, suggesting tumor-specific microbial signatures that may influence behavior and outcomes [96,97,98,99]. Reported enrichment includes *Actinomycetales*, *Acinetobacter*, *Streptococcus*, *Pseudomonas*, *Porphyromonas*, *Atopobium*, and *Peptoniphilus*, whereas non-tumor samples more often contained Bacillota and Actinobacteriota [96,97,98,99]. Some taxa inversely correlated with serum CA125 or segregate histology, proposing a candidate biomarker [97,98].

A recent systematic review indicates reduced alpha diversity in tumor relative to adjacent tissue, with lower Shannon and Chao1 indices and a modestly higher Simpson index, consistent with dominance by fewer taxa [99].

## 6. Antibiotics, Dysbiosis, and Therapy Resistance

Antibiotic exposure can reshape microbial communities with consequences for cancer progression and treatment efficacy. Population-based analysis associates antibiotic use near the time of cancer diagnosis with increased mortality across multiple malignancies, including OC, supporting antibiotic stewardship in oncologic care [100].

In high-grade epithelial OC treated with cytoreductive surgery and platinum chemotherapy, antibiotic exposure during chemotherapy has been linked to shorter progression-free and overall survival; anti-Gram-positive regimens appear particularly detrimental [101]. Mechanistic models align with these observations: high-dose antibiotics induced dysbiosis, accelerated OC growth, enhanced TNF-α and IL-6 secretion from tumor-associated macrophages, promoted EMT and angiogenesis, and expanded cancer stem-like cells; dysbiosis also exacerbates cisplatin resistance, whereas FMT from untreated donors restores chemosensitivity [80,102,103].

## 7. Microbiota and Treatment Response in OC

Standard treatment for OC includes cytoreductive surgery followed by paclitaxel-carboplatin chemotherapy [3]. Chemotherapy may partially restore gut diversity over time, enriching anaerobic taxa such as *Bacteroides*, *Collinsella*, *Faecalibacterium*, *Coprococcus*, and *Blautia*, while reducing opportunistic pathogens, including *Klebsiella* and other Enterobacteriaceae [104]. Preclinical models show that microbial integrity is required for optimal efficacy of platinum agents and innate-immune adjuvants, consistent with the *TIMER* framework (Translocation, Immunomodulation, Metabolism, Enzymatic degradation, and Reduced diversity) that summarizes microbiota-chemotherapy interactions [105,106].

**Figure 3:**
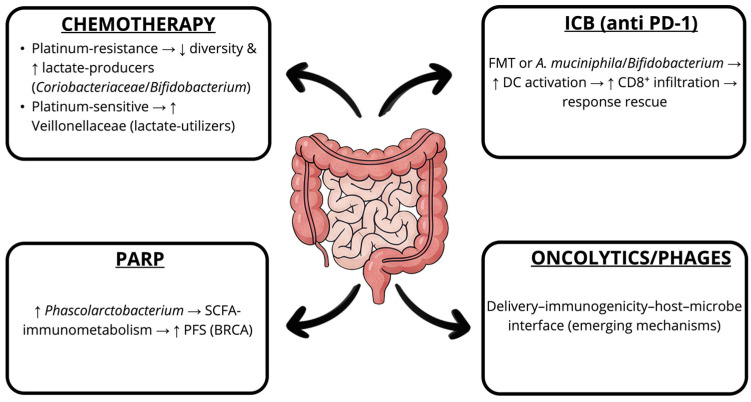
Gut microbial signatures modulate therapeutic efficacy in ovarian cancer. Gut microbial configurations associated with response vs. resistance to chemotherapy, immune checkpoint blockade (anti-PD-1), PARP inhibition, and emerging therapies. Microbial taxa shape antigen presentation, T-cell infiltration, and tumor immunometabolism, thereby modifying response and rescue potential across modalities. DC: dendritic cells; FMT: fecal microbiota transplantation; ICB: immune-checkpoint blockade; PD-1: programmed cell death-1; PFS: progression-free survival (PFS); SCFA: short-chain fatty acids.

In platinum-resistant OC, low-dose oral cyclophosphamide may be used palliatively; its activity depends on microbiota-mediated immune priming that reshapes the gut microbiota, permits Gram-positive translocation to secondary lymphoid tissues, and triggers Th17/Th1 responses essential for tumor control [107] (Figure 3).

In OC tissues, functional differences in KEGG (Kyoto Encyclopedia of Genes and Genomes) pathways may be more informative than taxonomic shifts alone, underscoring the need for multi-omics approaches [72].

Clinical and longitudinal cohorts reveal that platinum-resistant OC is associated with progressive loss of diversity and enrichment of lactate-producing taxa (e.g., *Coriobacteriaceae*, *Bifidobacterium*), whereas platinum-sensitive disease maintains more stable, diverse communities enriched in lactate-utilizing Veillonellaceae [86,104]. Together, these findings highlight the chemotherapy–microbiota interplay and its potential for therapeutic intervention.

Vaginal microbiota composition also correlates with treatment response, with *Lactobacillus*-dominant profiles less frequent in OC, particularly in platinum-resistance disease, while *Escherichia*-dominated communities associate with inflammation, poor prognosis, and shorter progression-free interval; dominance by *Lactobacillus iners* has been linked to minimal residual disease [108].

Checkpoint immunotherapy has not yet achieved durable benefits in OC; however, emerging OC-specific multi-omics data indicate a tumor–immune–gut axis associated with exceptional responses to PD-1 therapy [109]. FMT from responders restores PD-1 efficacy in mice, and supplementation with *A. muciniphila* or *Bifidobacterium* augments antitumor immunity by promoting dendritic cell activation and CD8^+^ T cell infiltration [110,111,112,113,114,115].

Targeted agents such as poly (ADP-ribose) polymerase inhibitors (PARPi) may also be modulated by the microbiome: higher fecal abundance of *Phascolarctobacterium* correlates with longer progression-free survival in BRCA1/2 wild-type patients, plausibly via SCFAs-mediated immunometabolic effects [116]. Emerging modalities such as bacteriophages and oncolytic viruses intersect with the microbiome through delivery, immunogenicity, and host–microbe interactions [117,118,119,120].

## 8. Therapeutic Potential of the Microbiome Modulation

Pharmacomicrobiomics extends pharmacogenomics by examining how host-microbiome interactions influence drug absorption, distribution, metabolism, excretion, efficacy, and toxicity [121,122,123]. Interindividual variability in microbial composition contributes to heterogeneous responses and adverse effects, suggesting opportunities for microbiome-informed precision oncology.

Probiotics (e.g., *Lactobacillus*, *Bifidobacterium*, *Saccharomyces*) may restore eubiosis (e.g., community structure and function comparable to healthy controls), regulate barrier integrity, and modulate immune responses [124]. Prebiotics, such as inulin and oligosaccharides, selectively enrich beneficial taxa [125]. Both approaches help restore microbial balance, produce metabolites such as SCFAs and vitamins, modulate immune responses, and influence drug metabolism [126]. Postbiotics, non-viable microbial cells or components, offer a favorable safety profile for immunocompromised hosts [40,127,128,129]. Despite promises in preclinical models, controlled OC clinical trials are lacking.

Diet is a principal determinant of microbiome composition and function. Mediterranean-style dietary patterns are associated with reduced all-cause and cancer mortality and may improve OC survival when adhered to before and after diagnosis, improving overall survival [130,131,132]. Intermittent fasting (16 h) has immunostimulatory effects in murine models that may be relevant to therapy synergy [133].

FMT has shown promise in oncology. In OC-bearing mice treated with cisplatin, FMT restored microbial balance, reduced tumor burden, repaired intestinal damage, decreased IL-6, and re-sensitized tumors to chemotherapy [83,103,110,114,134]. Vaginal microbiota transplantation is effective in recurrent bacterial vaginosis and merits exploration as a research tool for gynecologic oncology [135,136]. Microbiota also influences chemotherapy-induced toxicity, including cisplatin-associated cachexia and cardiotoxicity, where *Lactobacillus* supplementation mitigated damage and inflammation [137].

Given consistent signals that peri-treatment antibiotics correlate with poorer outcomes in OC, strategies that minimize unnecessary exposure and preserve microbial diversity should be prioritized, while prospective studies evaluate causality and mitigation approaches [100,101,138].

## 9. Challenges, Methodological Constraints, and Future Directions

Robust inference in microbiome-cancer research requires meticulous control for contamination, low biomass, and batch effects; standardized collection, extraction, and sequencing protocols; appropriate negative and positive controls; and multi-omics integration (metagenomics, metatranscriptomics, metabolomics, and host transcriptomics) [11,75,76,77]. Longitudinal designs and causal frameworks (e.g., Mendelian randomization, interventional trials) are needed to disentangle association from causation [92,93,94,95,139].

Large-scale initiatives, such as the ONCOBIOME consortium [140] are advancing the discovery of diagnostic, prognostic, and predictive microbial biomarkers. Early work integrating metagenomic profiling across the female reproductive tract suggests the feasibility of microbiome-informed risk stratification and therapeutic guidance in OC [140,141]. To accelerate it would be recommended: (1) consensus panels for specimen handling and sequencing; (2) harmonized clinical metadata; (3) pre-registered statistical plans; (4) cross-cohort replication; and (5) mechanistic validation.

The current evidence base is geographically narrow, with only a few studies of bacterial microbiota in OC from Denmark [142], Poland [143], India [144], China [69,72,80], and the United States [68,145,146], plus limited multi-center cohorts across Europe [55]. Methodological heterogeneity also constrains inference. Preclinical models from China and the United States [80,82,146] provide mechanistic insights, yet translational validation is scarce.

## 10. Conclusions

Microorganisms are emerging modulators of cancer biology across initiation, progression, and therapeutic response and may constitute a potential hallmark of cancer. In OC, converging evidence implicates gut and reproductive-tract microbiota in modulating tumor behavior and treatment outcomes. Nonetheless, high interindividual variability, late-stage presentation, and the scarcity of longitudinal multi-omics studies limit causal inference across the disease continuum.

With methodological standardization and rigorous mechanistic studies, validated microbial biomarkers and targeted microbiota interventions could make OC treatments more effective, less toxic, and more precisely tailored to patients.

## Data Availability

No new data were created or analyzed in this study.
